# The effect of pre-event instructions on eyewitness identification

**DOI:** 10.1186/s41235-023-00471-4

**Published:** 2023-02-28

**Authors:** Mario J. Baldassari, Kara N. Moore, Ira E. Hyman, Lorraine Hope, Eric Y. Mah, D. Stephen Lindsay, Jamal Mansour, Renan Saraiva, Ruth Horry, Hannah Rath, Lauren Kelly, Rosie Jones, Shannan Vale, Bethany Lawson, Josh Pedretti, Tomás A. Palma, Francisco Cruz, Joana Quarenta, Ine Van der Cruyssen, Mila Mileva, Jessica Allen, Brittany Jeye, Sera Wiechert

**Affiliations:** 1 University of Saint Francis, 2701 Spring Street, Fort Wayne, IN 46808 USA; 2 Oklahoma State University, 116 Psychology Building, Stillwater, OK 74074 USA; 3 Western Washington University, 516 High Street, Bellingham, WA 98225 USA; 4 University of Portsmouth, King Henry I Street, Portsmouth, PO1 2DY Hampshire UK; 5 University of Victoria, PO Box 1700, STN CSC, Victoria, BC V8W 2Y2 Canada; 6 University of Lethbridge, 4401 University Drive, Lethbridge, AB T1K 3M4 Canada; 7 Worcester State University, 486 Chandler St, Worcester, MA 01602 USA; 8 Swansea University, Singleton Park, Sketty, Swansea, SA2 8PP UK; 9 CICPSI, Faculdade de Psicologia, Universidade de Lisboa, Cidade Universitária, Alameda da Universidade, 1649-004 Lisbon, Portugal; 10 University of Amsterdam, 1012 WX Amsterdam, Netherlands; 11 University of Plymouth, Drake Circus, Plymouth, PL4 8AA UK

**Keywords:** Eyewitness identification, Lineup, Study instructions, Eyewitness memory

## Abstract

Research on eyewitness identification often involves exposing participants to a simulated crime and later testing memory using a lineup. We conducted a systematic review showing that pre-event instructions, instructions given before event exposure, are rarely reported and those that are reported vary in the extent to which they warn participants about the nature of the event or tasks. At odds with the experience of actual witnesses, some studies use pre-event instructions explicitly warning participants of the upcoming crime and lineup task. Both the basic and applied literature provide reason to believe that pre-event instructions may affect eyewitness identification performance. In the current experiment, we tested the impact of pre-event instructions on lineup identification decisions and confidence. Participants received non-specific pre-event instructions (i.e., “watch this video”) or eyewitness pre-event instructions (i.e., “watch this crime video, you’ll complete a lineup later”) and completed a culprit-absent or -present lineup. We found no support for the hypothesis that participants who receive eyewitness pre-event instructions have higher discriminability than participants who receive non-specific pre-event instructions. Additionally, confidence-accuracy calibration was not significantly different between conditions. However, participants in the eyewitness condition were more likely to see the event as a crime and to make an identification than participants in the non-specific condition. Implications for conducting and interpreting eyewitness identification research and the basic research on instructions and attention are discussed.

**Supplementary Information:**

The online version contains supplementary material available at 10.1186/s41235-023-00471-4.

## Significance statement

Eyewitness identification research has informed policy on how eyewitness identification procedures are conducted across the world. As we seek to use science to influence practice, it is important that this work be applicable to the legal field. An overlooked aspect of eyewitness identification research methods is the nature of the instructions that researchers give to their participants before exposing them to a mock crime and how much researchers reveal about upcoming tasks. In the real world, most eyewitnesses do not receive warnings or instructions before witnessing a crime. Despite this, researchers sometimes give warnings and instructions to participant witnesses in the laboratory. In this experiment, we found no effect of pre-event instructions on the ability of eyewitnesses to discriminate between guilty and innocent suspects in lineups or the usefulness of their confidence to predict their accuracy. However, we did find that those given eyewitness pre-event instructions were more likely to make an identification from a lineup regardless of its accuracy than those given non-specific pre-event instructions. Although more research is needed to be certain of the effect of instructions, our study suggests that revealing instructions are not a major threat to the applicability of eyewitness research that uses revealing instructions.

## Introduction

The literature on inattentional blindness demonstrates that if people are otherwise engaged they will not notice unusual things occurring in their vicinity such as a person walking by in a gorilla costume, money on a tree, or a crime occuring (Chabris et al., 2011; Hyman et al., 2014, 2018; Naka et al., 1996, Rivardo et al., 2011; Simons & Chabris, 1999; Simons & Schlosser, 2017). Nonetheless, people expect that they will notice such unusual events and are surprised when they and others fail to become aware of them (Levin et al., 2002). Whether a person notices and attends to a crime depends on both the circumstances of the crime and that person’s goals (Hyman et al., 2018). Conditions rarely align to cause a person to literally watch for a crime, as most people who witness a crime are likely engaged in an unrelated, personally relevant task and do not anticipate encountering a crime. In general, criminal activity may not engage our full attention immediately or at all (Hyman et al., 2018). Other than individuals employed as police officers, security guards, bank tellers, and convenience store workers, people do not usually receive instructions on what to attend to or a forewarning that they are about to witness a crime. When people do have some inkling or suggestion that a crime is about to occur, we still do not know much about what they may naturally do to prepare or how certain instructions might alter their cognition during the event.

Researchers sometimes try to create realistic conditions to examine memory for witnessed events (Darling et al., 2008; Douglass et al., 2005; Eisen et al., 2017; Phillips et al., 1999; Valentine et al., 2012; Wells, 1984). However, most researchers provide mock witnesses with a conspicuous simulated pre-recorded event, meaning that one or two actors perform clear actions within reasonable view and focus of the camera. As such, the laboratory setting involves far clearer task demands, a lack of distraction by concurrent tasks, and a much smaller field of view than most real-world eyewitnessing experiences. The simplicity of most of these pre-recorded events makes their witnessing conditions unlike those experienced by a majority of witnesses in the real world. Some researchers use even less realistic scenarios by providing pre-event instructions that either specify how participants should attend to the event (e.g., watch for a crime or criminal) or describe the tasks that will follow the event. When giving such informative and accurate instructions, researchers may unwittingly make events that may otherwise be inconspicuous, obvious to the participant or highlight the culprit, thus inflating eyewitness accuracy. Eyewitness identification researchers are thus faced with two problems. If some of the literature uses pre-event instructions and some does not use pre-event instructions, then there may be a hidden confound when comparing across studies. In addition, clear pre-event instructions also present an ecological validity problem in this field of research.

If pre-event instructions have an impact on eyewitness identification accuracy, then the eyewitness identification literature may overestimate eyewitness identification accuracy and choosing rates. Lab based estimates of eyewitness identification accuracy are used to draw conclusions about the reliability of eyewitness identification in the field, in which there are almost never explicit pre-event instructions. It is problematic if those conclusions are impacted by the use of pre-event instructions. Pre-event instructions may also affect eyewitnesses’ confidence-accuracy calibration, therefore the current study could reveal an unforeseen addition to the pristine conditions necessary to produce good calibration (Wixted & Wells, 2017). Finally, pre-event instructions may impact the effect of other important system and estimator variables on eyewitness identification. Pre-event instructions may reduce or eliminate the effect of poor eyewitnessing conditions such as exposure duration, biased lineups, or complex scenes, and they may produce crossover interactions with some variables. It is difficult to estimate the scope of these issues, both because pre-event instructions are rarely reported and any examples of non-significant results in this domain have likely gone unpublished.

In the current research, we manipulated pre-event instructions about the event and future memory tasks (i.e., lineup) using a conspicuous event. Hyman et al. (2018) established that clear pre-event instructions enable participant-witnesses to notice an inconspicuous event earlier and identify the culprit from a lineup more frequently. Though inconspicuous events are surely common in the real world, conspicuous events are more common in the eyewitness identification literature and thus merit a similar investigation.

Hyman et al. (2018) cautioned against generalizing about eyewitness memory based on studies in which participants know they are going to witness a crime, as this scenario has low realism and may skew estimates of eyewitness performance. Unfortunately, it is impossible to know the true frequency of the use of such instructions in the literature, as our systematic literature review (detailed below) found that pre-event instructions are rarely reported in detail. The goal of the current research is to test the effect of pre-event instructions on eyewitness identification to discover whether this difference between real-world witnessing and lab-based mock witnessing limits the generalizability of lab-based research. If pre-event instructions have a reliable effect on eyewitness accuracy, we will be faced with an emergent need to retest other variables known to affect accuracy to determine whether the impact of the variables are robust across changes in instruction.

To determine the types of pre-event instructions researchers have used in the extant literature, we collected and coded two groups of publications: those that influenced policy and those published recently. To account for influential publications, we collected the 278 papers cited in the most recent paper on policy recommendations for collecting eyewitness evidence in *Law and Human Behavior* (Wells et al., 2020). Two eyewitness memory researchers extracted papers pertaining only to eyewitness identification and lineups from the reference list, leaving 158 papers that qualified for inclusion in our list. Since the advent of the replication crisis in psychology (John et al., 2012; Simmons et al., 2011), practices have changed in many subfields. To account for the possibility that practices and reporting styles have changed in eyewitness identification research, we collected papers published during 2019 from research search engines. Specifically, we entered the exact same search terms ("eyewitness identification" or “lineup”)^1^ on PsychInfo, PsychArticles, and SCOPUS for papers published in 2019. In PsychInfo and PsychArticles, we limited the search to “peer-review” articles. In SCOPUS, we limited the search to “articles” in the subject areas of “Psychology”, “Social Sciences”, and “Neuroscience”. This literature search yielded 58 qualifying papers. We set further inclusion requirements: papers that contained at least one study, a witnessed event, and either a lineup or a showup identification task. Of these two sets of papers, 73 of Wells et al.’s (2020) cited papers and 23 papers published in 2019 met criteria for inclusion. Trained research assistants coded 102 studies from the Wells et al. (2020) citations and 42 studies from literature published in 2019. When there were multiple studies in a paper, they were coded separately. Two studies appeared in both sets, leaving a total of 142 to be coded. Though the rate of providing pre-event instruction varied somewhat between the two samples, the variation was not systematic and was not large enough to account for the major shift in reporting practices that the replication crisis has engendered, thus we see no compelling evidence that more recent reports were more likely to contain pre-event instruction information.

Pre-event instructions were broken down into two categories: attention/encoding instructions and instructions that informed participants of a future task. Overall, we found that pre-event instructions were not reported in most papers (see Table 1 for proportions, https://osf.io/zb85d/ for full database). We broke pre-event instructions down into two categories: reporting of instructions pertaining to attention/encoding and those pertaining to the future task. Approximately 74% of the 142 studies we coded did not include any details on their pre-event instructions that pertained to attention/encoding. Approximately 34.5% of the 142 studies we coded did not include any details on their pre-event instructions that pertained to the future task. Of the pre-event instructions that were reported, there was variability in both attention/encoding and future task pre-event instructions. The most commonly reported pre-event instructions were rather simple, including attention and encoding instructions such as “watch this video” or “pay attention to this video” (21.13% of all studies) and future task instructions such as “you’ll be asked questions about it later” (19.01% of all studies). Among instances where participants were told to pay attention to the video, they were also told something along the lines of “you’ll be asked questions about it later” approximately 57% of the time. Two of all of the coded studies (1.41%) reported telling participants to pay attention so that they could complete a later lineup task.

**Table 1:** Instruction Types by Source

	White paper					
Source	(*N* = 102)		2019 (*N* = 42)		Total (*N* = 142)	
	*n*	%	*n*	%	*n*	%
*Instructions and information before the witnessed event*						
Study details	7	6.86	6	14.29	13	9.15
Event details	13	12.75	3	7.14	16	11.27
Event instructions	28	27.45	18	42.86	46	32.39
Not reported	65	63.73	21	50.00	86a	60.56
*Attention instructions before witnessed event*						
Event	12	11.76	18	42.86	30	21.13
Crime	1	0.98	0	0.00	1	0.70
People	6	5.88	2	4.76	8	5.63
Target person	2	1.96	0	0.00	2	1.41
Other*	4	3.92	2	4.76	6	4.23
Not reported	83	81.37	24	57.14	105^a^	73.94
*Future task information before witnessed event*						
Asked questions	15	14.71	12	28.57	27	19.01
Memory task	1	0.98	1	2.38	2	1.41
Lineup task	2	1.96	0	0.00	2	1.41
Other*	5	4.90	1	2.38	6	4.23
Explicitly stated this was withheld	39	38.24	16	38.10	55	38.73
Not reported	40	39.22	11	26.19	49a	34.51
*Study description at recruitment*						
Eyewitness/forensic	3	2.94	6	14.29	9	6.34
Memory	7	6.86	3	7.14	10	7.04
Other*	30	29.41	5	11.90	53a	37.32
Not reported	66	64.71	30	71.43	96	67.61

^*a*^Total column does not double-count the 1 paper that appears in both sets

Some studies used more than one instruction type and are counted more than once, percentages are based on actual total number of papers

^*^“Other” category in Attention Instructions included instructions to watch for suspicious behavior or to focus on conversations and non-verbal behavior. “Other” category in Pre-Crime Future Task Information included telling participants that they would later give a verbal description of the perpetrator, that they would later work with a sketch artist to create a composite, that they would later “give evidence,” that they would give their impressions and reactions to the film, that they would give their impressions of the people in the film, or that researchers would examine the effects of exposing the participant to the film. “Other” category in Study Description at Recruitment included telling participants the study was about: Impressions of People, Perception, Personality, Personality and Perception, Perceptions of a Speech, Impressions after viewing people, Group Interactions, quality of campus security video, Subliminal Perception, Psychology and Education, Artistic Quality of Film, Information Processing, Video Game Performance, Biofeedback Demonstration, and Alcohol on Cognitive and Motor Functions

Related to the issue of revealing pre-event instructions is the information given to participants during recruitment (in some cases, the cover story of the study). Just over half (51%) of studies contained information about the cover story used during recruitment. Of those that reported recruitment information, some reported informing participants that the study was about eyewitnesses or forensic psychology (*n* = 9, 6.34% of all studies) or that there would be a memory test later (*n* = 10, 7.04% of all studies). Thus, some participants knew or could have inferred that their memory would be tested or even that they would be completing a lineup before they witnessed the event.

The dataset generated by our review was quite rich, and two main observations emerged. First, pre-event instructions were generally completely unreported. Second, there was wide variation in the amount revealed about eyewitness identification studies before participants witnessed the events amongst the small minority of studies that did report any details. Unfortunately, the question of whether the same pattern of results would emerge if every paper reported their pre-event instructions remains unanswerable because of the generally low rate of reporting these details in the current literature.^2^ Pre-event instructions are a source of uncontrolled and unreported variation in eyewitness identification studies that may impact performance. If pre-event instructions impact performance, studies featuring crime specific pre-event instructions that direct attention or reveal future tasks generalize to reality less than previously expected. This lack of generalizability may have implications for interpreting existing eyewitness identification studies and may call into question evidence for important findings and theories. One such finding is that confidence is highly predictive of accuracy *as long as* confidence is assessed under *pristine conditions,* or when identification procedures (i.e., system variables) are done using best practices (Wixted & Wells, 2017).

Concern about the effect of pre-event instructions is based on the effects of instructions on attention, encoding strategy, and metacognition reported in the basic memory literature. Basic research has found that intentional encoding impacts the orientation of attention (Varakin & Hale, 2014) and the level at which participants process and remember material (Craik & Tulving, 1975), especially faces (Coin & Tiberghien, 1997). When trying to encode material, people often adopt intentional encoding strategies and are more likely to engage in rehearsal than when they are not trying to encode material. Other basic work indicates that instructions may impact metacognition. For example, judgments of learning varied depending on whether participants received incidental or intentional encoding instructions (Mazzoni & Nelson, 1995). Cox et al. (2021) found that instructions changed performance in a lower-level visual search task, which they hypothesized was due to a change of expectation of target frequency. To bridge the gap between basic and applied research, Shapiro and Penrod (1986) meta-analyzed 128 face memory studies, 20% of which were eyewitness identification studies. In a subset of those studies (*n* = 29), encoding instructions to make inferences about the personality of the face caused more hits and a somewhat lower false alarm rate. But we do not know what proportion of this subset of studies were eyewitness identification studies. Mansour et al. (2017) examined the impact of administering multiple lineup paradigms to participants on eyewitness identification and confidence. The instructions provided to participants were not manipulated, instead the researchers were interested in whether experience would impact participants' approach to and thus performance on the task. The researchers found that experience positively impacted correct identifications, but the effect size was small. Pre-event instructions may have a larger effect on eyewitness identification because they are more overt and explicit than experience which requires metacognition and reflection.

Applied researchers have rarely tested how pre-event instructions affect lineup identifications specifically, and their results have been mixed. Cowan et al. (2014) did not use pre-event instructions per se but did warn half of their participants of a forthcoming lineup at the midpoint of their witnessed event. They explicitly advised participants to engage in activities to enhance their lineup accuracy. After a two-week delay, the warning enhanced lineup accuracy but did not have an effect on lineup confidence. Lindsay et al. (1998) found that participants who got a good view of the culprit and were told that they would later complete a lineup had higher identification accuracy rates and higher confidence than participants who got a poor view and were told they would be asked to identify the filming location of the video. However, Lindsay et al. did not separate instructions from viewing conditions. Both sets of researchers found evidence that instructions about an upcoming lineup impact eyewitness identification, though neither provide an explicit manipulation of pre-event instructions.

Other researchers found that certain types of pre-event instructions did not affect lineup performance. Like Cowan et al. (2014), Yarmey (2004) did not use pre-event instructions but manipulated whether participants were told that it was important to remember a target’s face in the midst of an interaction with the target. Yarmey found no differences in lineup identification between individuals who were told it was important to remember a target’s face compared to those who were not, but they did find some evidence that instructions enhanced recall of physical and clothing characteristics.

Wulff and Hyman (2022) manipulated pre-event instructions in a crime blindness study. Crime blindness refers to inattentional blindness for a crime, wherein a person does not notice a crime though it is available to be noticed in their visual field (Hyman et al., 2018). Wulff and Hyman tested the prevalence of crime blindness through showing participants a 1 m, 48 s video in which many actors enter and exit the frame throughout a busy university hallway scene in which a man steals a backpack (at 1:12). Wulff and Hyman’s video features a crime that is not the focus of the event among several other actors milling about the scene. Participants were told to watch the video (control condition), to count the number of people wearing white (inattentional blindness condition), or to watch for a theft (eyewitness condition). The comparison between the control condition and the eyewitness memory instruction condition is of import to the current study. One hundred percent of participants in the eyewitness memory instruction condition (i.e., “Watch for a theft.”) noticed the crime, whereas only 61% of those in the control condition (i.e., “Watch this video.”) noticed the crime. Identification of the perpetrator did not vary by instruction, but participants in the eyewitness instruction condition were more likely to incorrectly identify an innocent bystander in the lineup task in which both the perpetrator and the bystander were present. However, as the lineup analysis was not the primary measure it may have been underpowered and most laboratory studies of lineup accuracy use stimuli different from Wulff and Hyman’s video.

An equally important issue to accuracy is how pre-event instructions impact confidence in an identification. If accuracy and confidence are well calibrated then confidence can be used as a marker of accuracy in criminal cases. Confidence and accuracy are generally well calibrated, but eyewitnesses tend to be overconfident in their accuracy (Brewer & Wells, 2011). Wixted and Wells’ (2017) reanalyses led them to conclude that confidence is highly predictive of accuracy if the identification occurs under pristine conditions. Researchers have publicly commented to the courts that high confidence is associated with high accuracy (Fikes, 2015), which impacts perceptions of eyewitness evidence in court and the odds of conviction. Pre-event instructions may enable participants to have better witnessing conditions and to be more aware of them, which would lead to better calibration between accuracy and confidence. The cognition research demonstrating that instructions impact attention orientation and encoding strategy suggest that pre-event instructions may enable participants to improve their performance on a lineup task. Mazzoni and Nelson (1995) found that people’s judgments of learning were more accurate after intentional encoding than after incidental encoding. If pre-event instructions lead to better confidence-accuracy calibration, then pre-event instructions may be a heretofore unconsidered pristine condition.

Researchers have discovered boundary conditions or exceptions to the specifications of high confidence-accuracy calibration made by Wixted and Wells (Colloff et al., 2016; Giacona et al., 2021; Grabman et al., 2019; Lockamyeir et al., 2020; Seale-Carlisle et al., 2019; Semmler et al., 2018). For example, when multiple estimator variable conditions are poor, high confidence identifications are less reliable no matter how unspoiled the identification conditions (Giacona et al., 2021). Giacona et al. (2021) suggested that people may not have strong enough metacognitive knowledge to appropriately calibrate their confidence to their identification decision. Overconfidence is exacerbated when participants are given biased lineup instructions (Brewer & Wells, 2006) and in other situations (Sauerland et al., 2019). As biased lineup instructions and pre-event instructions are both instances of eyewitnesses being given potentially useful information before they begin the memory task, informative pre-event instructions may also lead to overconfidence without a concomitant increase in accuracy relative to no instructions. Examining the impact of pre-event instructions may help to reconcile discrepant findings in the literature and will help to obtain a more realistic estimate of the confidence-accuracy relationship in eyewitness identification.

The existing studies provided only one type of instructions (either attention or future task) and either issued instructions during the event, manipulated instructions in a confounded way to test a higher-order variable such as “witness quality,” or manipulated instructions outside of the context of a typical eyewitness paradigm. In the current research, we (a) issued pre-event instructions that will orient participants’ attention to the crime and reveal an upcoming lineup before the start of a video, (b) showed a video with a conspicuous event depicting only the criminal, and (c) systematically manipulated instructions. With all these issues satisfied, we present a controlled and strong test of the effect of instructions on eyewitness identification and the confidence-accuracy relationship.

## The current experiment

Existing studies typically do not report the pre-event instructions that they use and those that are reported vary. In addition, we do not yet have a strong understanding of the impact of pre-event instructions on eyewitness identification, which could impact the interpretation and generalization of the existing literature. In the current experiment, we sought to examine whether pre-event instructions about the event and future tasks impacted eyewitness identification accuracy. We aimed to do so using eyewitness identification materials and procedures that reflected those commonly used in the literature to draw conclusions that generalized to the literature. Most studies in this field, including most studies cited by Wells et al. (2020), use events featuring easy to detect crimes with clear views of the criminals involved. We do not yet know what impact pre-event instructions might have on the conclusions drawn from studies using this methodology. We tested a strong manipulation of pre-event instructions to search for a basic effect. Participants in the eyewitness condition were informed that the video would depict a crime and that they would later be tested on their ability to identify the culprit in a photospread lineup. Participants in the non-specific instruction condition were simply told “Watch this video.” We predicted that participants in the eyewitness condition would have better discriminability and thus produce a Receiver Operating Characteristic curve (ROC curve) with more area under the curve than participants in the non-specific condition. We also hypothesized that participants in the eyewitness condition would be more overconfident. Regarding confidence-accuracy calibration, we hypothesized that eyewitness instructions may lead to better calibration if participants can use the instructions to inform their study of the event and metacognitive beliefs about what they’ve witnessed. Alternatively, eyewitness instructions may lead to worse calibration if participants are not able to improve their study of the event or if their metacognitive abilities are not strong enough to lead to proper calibration. In addition, we collected self-report data that addressed the difference between expecting a crime and knowledge of a future task (including questions about awareness of the crime, attention paid to the video, and intentions while watching the video), which we hypothesized would be impacted by instructions and predict lineup identification accuracy.

## Method

### Participants

Participants were recruited to participate online; for class credit using participant pools across several universities, or for compensation from crowdsourcing professional participant pools (i.e., TurkPrime and/or Prolific). Data were collected across several universities through an organization called the Eyewitness Undergraduate Research Consortium, run by a co-author (similar to the Many Labs approach, i.e. Klein et al., 2014). Participants were 18 years of age or older and self-reported speaking fluent English. They completed a set of demographic questions including ethnicity, for later logging of cross-race identifications.

Our primary outcome measure was a partial Receiver Operating Characteristic (pROC; Mickes et al., 2012), designed specifically for eyewitness identification performance.^3^ Colloff and Wixted (2020) cited a range of sample sizes in previous lineup pROC studies from 300 to 500 per condition. By converting Wetmore et al.’s (2015) test statistic into a measure of standard error, Colloff and Wixted concluded that 500 participants per condition would offer 80% power to detect an effect of the same size found by Wetmore et al. (2015) in the partial lineup ROC procedure (Mickes et al., 2012).^4^

We also constructed full lineup Receiver Operating Characteristics (full ROCs; Smith et al., 2020a, 2020b) to identify possible differences between analyses with and without filler IDs. Full ROC curves presumably require fewer participants as all those who choose a filler member of the lineup enter the ROC calculations. The medical literature informed our sample size decisions. Medical researchers using ROC procedures akin to the full lineup ROC recommend that sample size be based on the sensitivity (correct ID rate / (correct ID rate + miss rate)) and specificity (correct rejection rate / (correct rejection rate + false ID rate)) of the test, as well as the prevalence of the signal (# of CP lineups shown / total # lineups shown), which leads to an estimate of 241.6 participants per condition based on our pilot data (see Pilot Study folder and Full ROC Sample Size Calculator in Files section of https://osf.io/zb85d/; Baratlook et al., 2015; Buderer, 1996; Negida et al., 2019). Thus far, the only published use of this method is Lampinen et al.’s (2020) recent test of pre-lineup instructions, in which they found no significant differences between groups with just under 500 participants each.

In the Stage 1 Report, we planned to test the hypotheses using pROC at prespecified points using sequential analyses (Lakens, 2014), namely after the collection of 250 and 500 participants per group. The medical literature recommendations guided our first stopping point and Lampinen et al. (2020), Colloff and Wixted (2020), and Wetmore et al. (2015) guided our final sample size goal. Likewise in the Stage 1 Report, we set the alpha level for all hypotheses to 0.029 using the Pocock boundary based on conducting the analyses 2 times (Pocock, 1977). If we found a difference between the area under our lineup pROC curves after collecting data from 250 participants per group, we planned to terminate data collection. If not, data collection would continue until we achieved our final prespecified sample size (500 per group). After collecting 250 participants per group, we constructed ROC curves and found bins with sample sizes below 5 (see student conference presentation on OSF, Pedretti et al., 2022), which was not enough participants per bin to build pROC curves with reliable estimates of accuracy at each confidence level. We thus could not perform a test using the pROC package, so we decided to collect data to the full sample size of 500 per group and dropped the Pocock alpha level adjustment because we did not conduct the previously planned sequential analyses (Mickes et al., 2012; Xavier et al., 2011). At the end of data collection, we had data from 1346 participants, which was reduced to 1149 after the exclusion criteria described below. We thus slightly overshot our intended sample size, as it is difficult to precisely control sample size when collecting data across many labs. We elected to include all data collected before the cutoff date decided by the co-authors.

### Design

We manipulated pre-event instructions as a between-subjects variable. Participants were given non-specific (i.e., “Watch this video”), or eyewitness (i.e., “Watch this video of a crime. You will be asked if you can identify the criminal from a lineup later”) pre-event instructions. The non-specific instructions served to orient attention very generally whereas the eyewitness instructions oriented attention to the crime and alerted participants about the future task. In addition, half of participants saw a culprit-present lineup and half saw a culprit-absent lineup. Our primary measures of interest were eyewitness identification performance and confidence.

### Materials

#### Event videos

We selected an exposure duration of 5 s based on our own previous work and Palmer et al.’s (2013) exposure times of 5 s and 90 s. The relatively shorter exposure duration maximized the odds of detecting any effect of pre-event instructions under conditions where the eyewitness had a clear view of the culprit. To reduce the risk of stimulus specificity effects, there were two different versions of the same crime video each including one culprit, 4 s of exposure, and 5 s in length (cut down from 41 s videos). The two culprits were description-matched (Caucasian, light brown/blonde hair, medium build, ~ 20 years old). The videos featured the culprit stealing the same car and were recorded in high definition on a university campus in the Pacific Northwest for a previous study (see https://osf.io/zb85d). In both videos, a man enters an office, takes keys from a desk, walks across a parking lot, finds a car, and unlocks the car with the stolen key. The video then shows him getting into the car and starting the engine. The man is onscreen throughout the video and is the only person shown. When his face is not visible, the view is typically of the back of his head, the desk, or the car.

#### Lineups

Photographs for the lineup were taken from multiple face databases created by or in the labs of one or more of the authors. Each person’s face was captured looking directly into the camera. Photos were cropped at the neck to remove any cues from clothing, and both culprits matched their appearance from the video (same haircut, no major face shape changes). Photographs of Caucasian men with blonde or light brown hair were pulled from the databases, and those that the first and second author agreed matched each culprit’s appearance best were placed in a six-member lineup for each culprit. The individual photos were approximately 371 × 383 pixels in size, and lineups were pre-tested to ensure performance was not at ceiling (Table 2). These materials can be found on the Open Science Framework (OSF) page for the study for which they were originally designed (https://osf.io/b8tk9/). We pilot tested the lineups for fairness two ways: by presenting a description alongside a lineup, and by presenting the crime video with pre-event instructions that described the forthcoming lineup task. Even with these easy and clear instructions, performance was not at ceiling (see Table 2), and Tredoux’s *E* and functional lineup size indicated high fairness for both lineups in both pilot tests (see Table 2). The filler chosen most often in the video exposure pilot study was designated the innocent suspect for each CA lineup.

**Table 2:** Proportions of response type by lineup, pilot study

Presentation	Lineup	Suspect IDs	Filler IDs	Rejections	Tredoux’s E (95% CI)	Functional Size
Video	Culprit present 1	10	11	4	4.63 (2.91, 11.31)	2.1
	Culprit present 2	11	7	5	3.73 (2.28, 10.18)	1.63
	Culprit absent 1	10	13	2	3.6 (2.52, 6.31)	2.3
	Culprit absent 2	10	11	4	3.65 (2.46, 7.05)	2.1
Description	Culprit present 1	12	39	NA	4.46 (3.64, 5.76)	4.25
	Culprit present 2	7	43	NA	5.48 (4.62, 6.74)	7.14
	Culprit absent 1	10	40	NA	5.51 (4.99, 6.15)	5
	Culprit absent 2	3	48	NA	4.07 (3.16, 5.72)	17

For culprit present lineups, suspect IDs are correct. For culprit absent lineups, rejections are correct

### Procedure

This protocol was approved by both the first authors’ university human research ethics committees and by research ethics committees at all Consortium institutions that participated in data collection. Participants were invited to complete our study under the name “Perceptions and Cognition”. Participants learned that the study concerned human cognition and that they would see images or a video and may be asked questions about them. The full recruitment statement and consent form, which contains additional details that participants will learn about the study before it begins, are available on OSF (https://osf.io/zb85d/). Participants were randomly assigned to receive one of two pre-event instructions: non-specific (i.e., “Watch this video”), or eyewitness (i.e., “Watch this video of a crime. You will be asked if you can identify the criminal from a lineup later.”). The instructions appeared on the screen as a screenshot of text. On the page displaying the instructions, participants were required to type the instructions in an open-ended response space to show they had read every word. Displaying the instructions as a screenshot prevented participants from copying and pasting the text, and the page did not advance until they entered the instructions exactly as written. Participants then watched a randomly assigned video, answered two attention check questions, completed a filler task (15 trials of simple mental rotation items), and then were presented with a lineup. Participants were randomly assigned to see either a culprit-absent (CA) or culprit-present (CP) lineup. They were informed of the option to reject the lineup and asked to rate their confidence immediately after making their identification decision.^5^ We asked a set of post-task debriefing questions (Table 3) to determine what participants thought the video was about and how they attended to the video. Specifically, we assessed participants' memory for the video, their perceptions of whether and when a crime occurred, participants' expectations of the event, what they tried to memorize, amount of attention paid to the video, and what strategies they used while watching the video.

**Table 3:** Debriefing questions

Question	Question type	Answer options
Before the video started, what did you think the study was about?	Open-ended	
What happened in the video?	Multiple choice	Guy grabbed his car keys and went to leave work, Guy grabbed keys off a desk and stole the car they were for, Guy argued with a friend, Don’t know
At what point in the video did you know a crime was happening?	Multiple choice	When he steals the keys, In the final moments when he starts the car, Never
Which of these were the instructions you received BEFORE the video?	Multiple choice	Watch this video, Watch this video of a crime. You will be asked if you can identify the criminal from a lineup later., I can’t remember
Before the video started, did you believe the video would feature a crime? Note that we aren't asking whether you think the crime was real, just whether you believed that a crime would be shown	Multiple choice	Yes, No
If no, why not?	Open-ended	
Before the video started, did you expect to see a lineup after the video?	Multiple choice	Yes, No
Did you alter the way you would have watched the video because of your instructions?	Multiple choice	Yes, No
If yes, How?	Open-ended	
Did you specifically try to memorize the man's face?	Multiple choice	Yes, No
If yes, at what point in the video did you make this decision to memorize his face?	Multiple choice	In the final moments, when he starts the car; when he leaves the office; when he steals the keys; as soon as he rounded the corner; before the video began; other (textbox)
Did you specifically try to remember other details from the video?	Multiple choice	Yes, No
If yes, list the details you tried to memorize here	Open-ended	
Is there anything else you'd like us to know about your experience in this study today?	Open-ended	
Please rate your attentiveness through the course of this study	Multiple choice	Very inattentive, fairly inattentive, fairly attentive, very attentive
Because you are completing this study online and on your own, some creative but unintended methods may have been tempting. Did you:	Multiple choice	Take a screenshot of the video to use later or open it in a side window to look at later, Pause the video to study details or watch it more than once, Neither of these

## Results

### Data storage

Raw de-identified data, analysis code, pilot data, and the Stage 1 Registered Report manuscript are available on the OSF at osf.io/zb85d.

### Exclusion criteria

Two attention-check questions were asked after participants viewed the crime video. If a participant answered both of these questions incorrectly, their data were excluded (*N* = 24). Data from participants who admitted to being inattentive (*N* = 6) or to cheating (N = 41) were also excluded. Data from participants who answered the identity question (i.e., What is your favorite food?) inconsistently across different instances were also excluded (*N* = 10).^6^ Cross-race identifications were not excluded. Some participants’ (*N* = 3) open-ended responses included admissions of internet or other issues that caused them to not be able to watch the whole video; these data were not removed because we did not identify this source of removal a priori, they add realistic variability to the dataset, and there are too few to meaningfully change any groupwise outcome measures. In addition, 127 participants who started the survey but did not complete it and 1 participant who did not provide a lineup confidence judgment were excluded. After exclusions, the final sample consisted of 1149 participants, 580 in the eyewitness condition and 569 in the non-specific condition.^7^

### Data analysis

#### Raw data descriptives

We tested whether overall identification accuracy, confidence, and the frequency with which the participant chose somebody from the lineup (i.e., choosing) varied by pre-event instruction condition; see Table 4 for means. As this set of analyses involves three independent t-tests and one between-subjects ANOVA, we adopted a per-test Bonferroni-corrected alpha level of 0.0125. Accuracy and confidence level did not significantly differ between conditions, *t*(1147) = 2.12, *p* = 0.034, and *t*(1147) = − 2.12, *p* = 0.034, respectively. We conducted a between subjects t-test on participants’ choosing rates, which showed that participants made a lineup selection more often in the eyewitness condition than participants in the non-specific condition, *t*(1147) = 2.97, *p* = 0.003, *d* = 0.18 (95% CI [0.06, 0.29]). This somewhat unexpected significant finding led us to conduct an exploratory ANOVA testing the effect of instructions conditions and culprit presence on choosing rates. The ANOVA showed the same effect of instructions, *F*(1,1145) = 8.89, *p* = 0.003, *η*^2^ = 0.008, but no effect of culprit presence, *F*(1,1145) = 0.36, *p* = 0.546, *η*^2^ = 0.0003, and no interaction effect, *F*(1,1145) = 1.15, *p* = 0.284, *η*^2^ = 0.0009.

**Table 4:** Response counts

Instructions	Culprit presence	Suspect IDs	Suspect ID avg confidence	Filler IDs	Filler ID avg confidence	Rejections	Rejection avg confidence	*N*
Eyewitness	Culprit absent	31 (10%)	3.02	204 (68%)	3.39	63 (21%)	3.32	298
	Culprit present	78 (28%)	4.00	141 (50%)	3.48	63 (22%)	3.59	282
Non-specific	Culprit absent	34 (12%)	3.09	157 (56%)	3.43	88 (32%)	3.00	279
	Culprit present	82 (28%)	3.50	129 (44%)	3.32	79 (27%)	3.22	290

#### ROC analysis

To test our hypotheses about discriminability and response bias, we broke down the raw data based on signal detection theory (SDT) as applied to eyewitness identification research. To create an ROC curve, one must first aggregate all responses by confidence level; cumulative hit rates are then plotted against cumulative false alarm rates at each level of confidence. The leftmost point on the curve represents hits and false alarms made with the highest level of confidence. The rightmost point includes cumulative identification rates across all levels of confidence. Deriving the area under these curves then provides a single measure of discriminability to compare between conditions with a t-test.

**Fig. 1:**
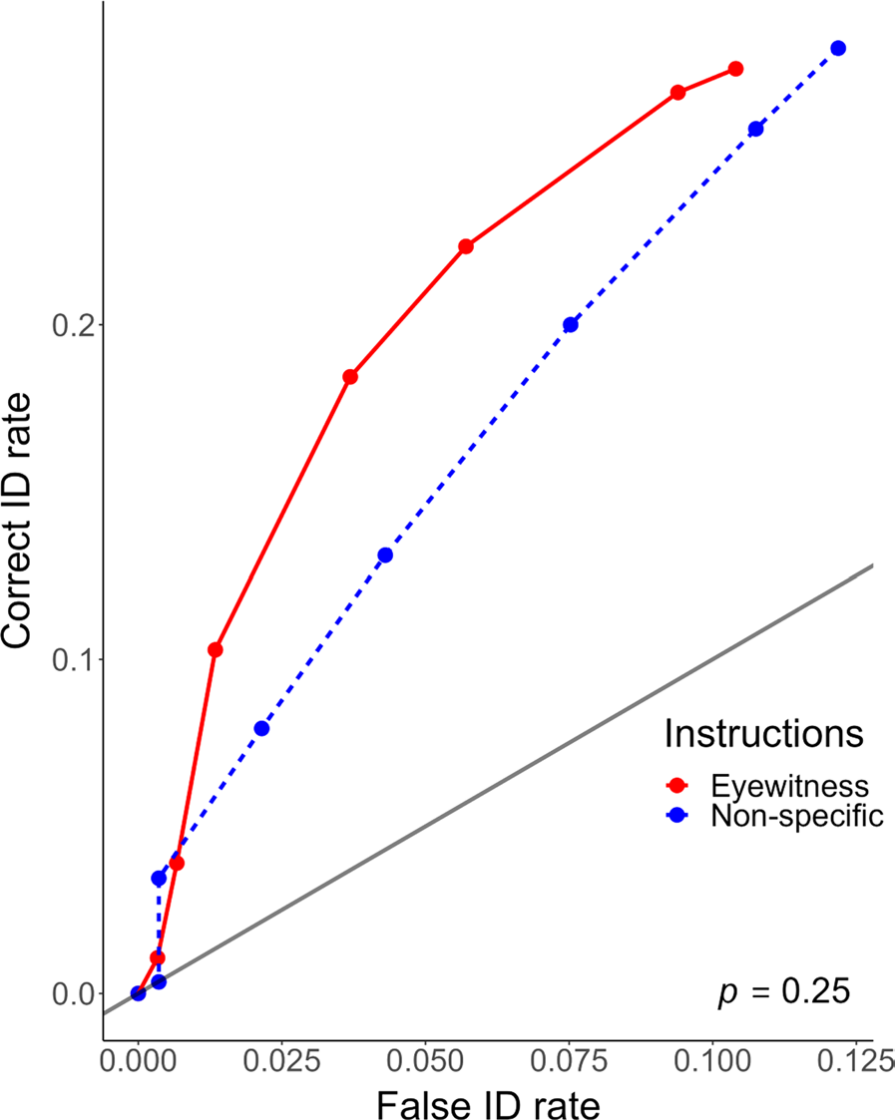
pROC

Figure 1 shows partial ROC (pROC) curves for each pre-event instruction condition, which are plotted from only suspect identifications (Gronlund et al., 2014; Mickes et al., 2012). Because pROC curves are truncated on the x-axis (FA rate), we cut off our measurement of the area under the pROC curve (pAUC) at the lowest observed false alarm rate (0.10). The resultant pAUC values were compared using the pROC package (Xavier et al., 2011) for R (R Core Team, 2022). The bootstrapped pAUC for the non-specific condition was 0.015 (95% CI [0.01, 0.02]). The bootstrapped pAUC for the eyewitness condition was 0.020 (95% CI [0.014, 0.026]). There was no statistically significant difference in discriminability as measured by pAUC between conditions, *D* = 1.15, *p* = 0.25.

**Fig. 2:**
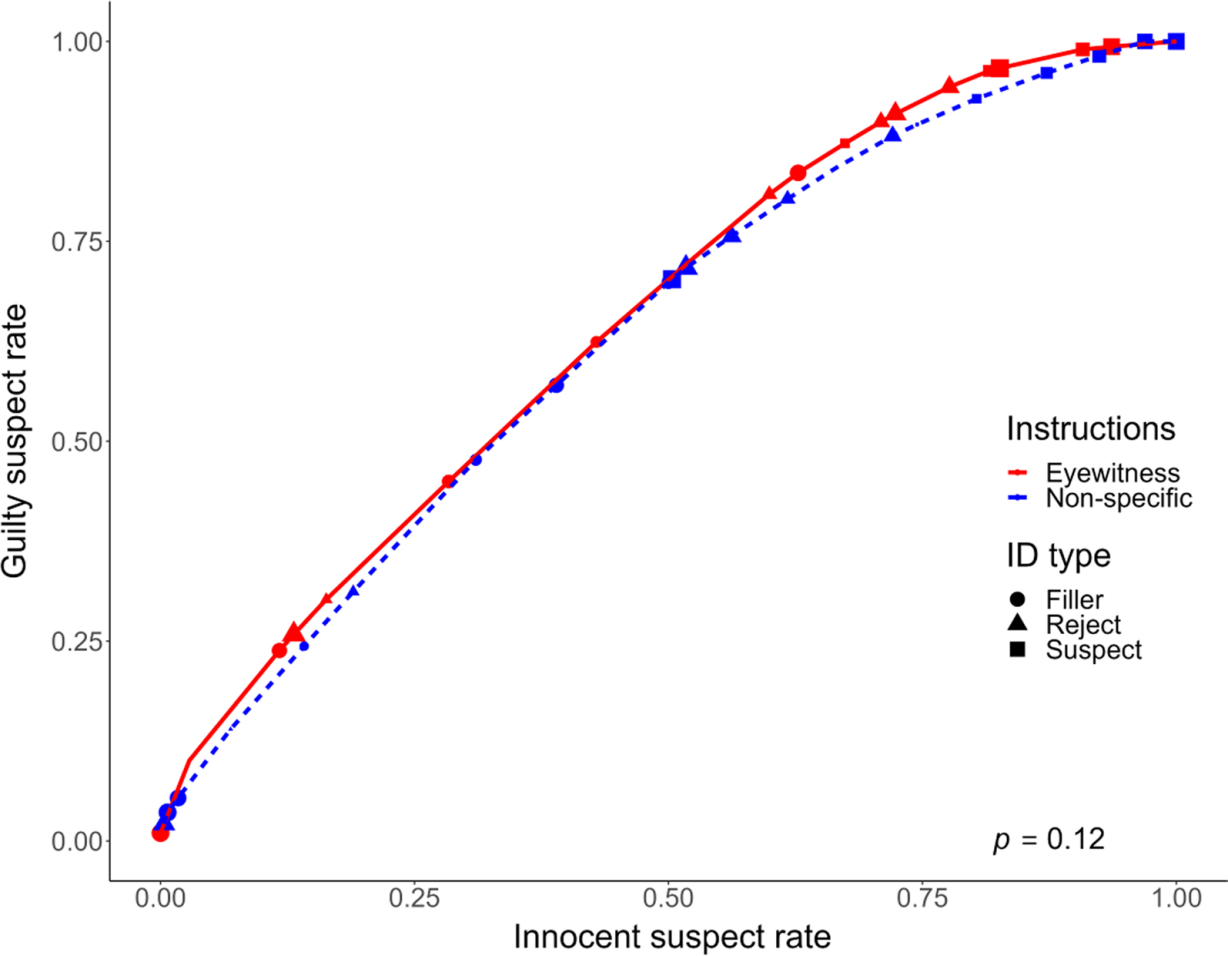
Full ROC

We also constructed and compared full ROC curves (as per Smith et al., 2020a; Smith et al., 2020b; using the fullROC package for R; Yang & Smith, 2022), which are shown in Fig. 2. Full ROC curves take into account the inculpatory/exculpatory evidentiary value of filler picks and rejections (from the perspective of the investigator), and therefore include all decision types as opposed to just suspect identifications. The bootstrapped full AUC for the eyewitness condition was 0.59 (95% CI [0.54, 0.64]), compared to a full AUC of 0.53 (95% CI [0.49, 0.59]) in the non-specific condition. A bootstrap comparison of these AUCs was not statistically significant (*p* = 0.12).

#### Confidence-accuracy relationship

**Fig. 3:**
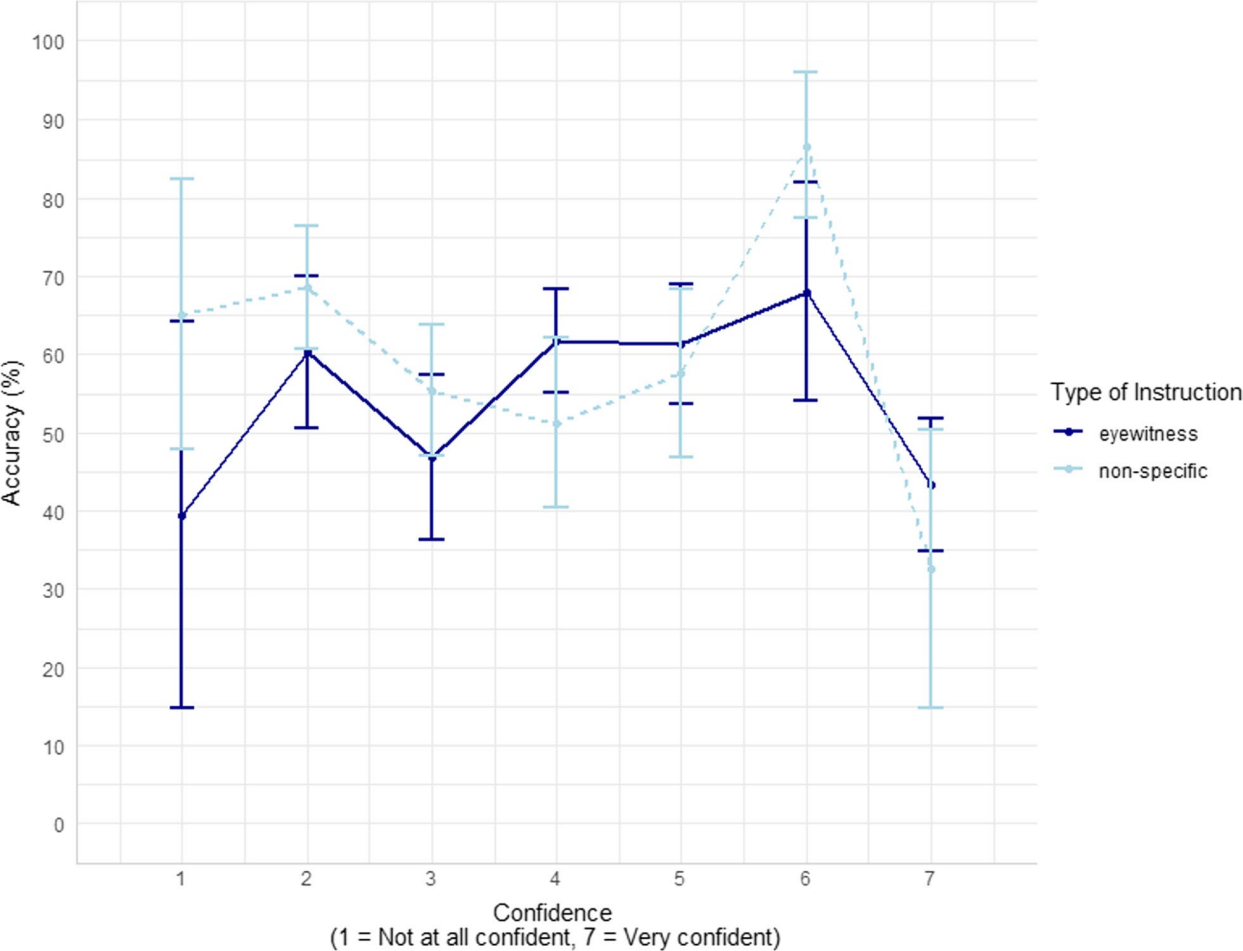
7-bin CAC

**Fig. 4:**
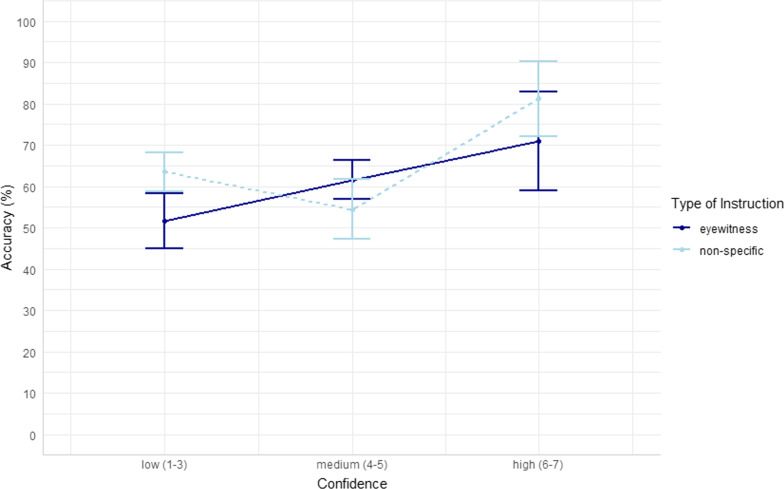
3-bin CAC

To test our hypotheses about confidence, we report Confidence-Accuracy Characteristic analyses (CAC; Mickes, 2015; Seale-Carlisle et al., 2019). To conduct the confidence-accuracy analyses we used the following R packages: here (Muller & Bryan, 2020), readxl (Wickham & Bryan, 2022), dplyr (Wickham et al., 2022), r4lineups (Tredoux & Naylor, 2018), boot (Canty & Ripley, 2021; Davison & Hinkley, 1997), ggplot2 (Wickham, 2016), psych (Revelle, 2022), and tidyverse (Wickham et al., 2019). While ROC curves assess discriminability, CAC curves assess the trustworthiness of an eyewitness’s confidence in their identification decision, which varies independently of discriminability. As the intention of this measure is to inform policymakers and triers of fact, we implemented a method suggested by both Smith et al. (2020a) and Fitzgerald (2020), in which the total number of false identifications in a condition is divided by the lineup’s functional size (as measured by Tredoux’s *E*). We calculated *E* and divided overall identification rates (hits / foil IDs + rejections) by *E* for each confidence bin. The *E* values for the lineups were 3.61 and 4.74. As per Fitzgerald (2020), we refer to this method as the creation of CAC/*E* curves. When split by all conditions in the experiment, some confidence level bins in the 7-bin CAC curve were left with very few observations, which resulted in very wide error bars around those estimates (see Fig. 3). We thus determined this analysis to be inconclusive, and collapsed the data into wider confidence bins: low confidence (ratings of 1–3), medium confidence (ratings of 4–5) and high confidence (ratings 6–7).^8^ The 3-bin CAC/*E* curve is shown in Fig. 4, with standard error bars for the CAC plots bootstrapped according to Seale-Carlisle and Mickes (2016). As these curves are somewhat new in the literature, we constructed traditional CAC curves using using our predesignated innocent suspect and found that the results were largely consistent with the results of the CAC/E curves (See Additional file 1). Regarding whether high confidence was related to high accuracy, we had a small number of cases (Eyewitness N = 13; Non-specific N = 11) at our highest confidence levels 6–7, therefore conclusions about high confidence from our data may not be reliable.

After the Stage 1 Report, we became aware of other methods of analyzing confidence data from Boekaerts and Rozendaal (2010)^9^ and a method of comparing *OU* (over/under-confidence) values with inferential confidence intervals (Tryon, 2001), thus we did not conduct chi-square analyses as written in the Stage 1 manuscript. The calibration statistics we calculated (3 bins) were *OU,* which indicates the extent to which, across the different levels of confidence, participants were overconfident (were more confident than they were accurate) or underconfident (less confident than they were accurate), calibration (*c*) which indicates how well calibrated the participants’ confidence was overall, and the adjusted normalized discrimination index (*ANDI*; Yaniv et al., 1991) which reflects how effectively confidence discriminates between accurate and inaccurate eyewitness identifications in the sample. We considered over/underconfidence for participants in the eyewitness instructions condition (3 bins): OU = − 0.02, 95% CI [− 0.18, 0.12] and for participants in the non-specific instructions condition (3 bins): OU = − 0.10, 95% CI [− 0.30, 0.14]. We calculated 95% inferential confidence intervals (ICIs) for *OU* and found that they overlapped, indicating no significant difference (Eyewitness condition: [− 0.16, 0.12]; Non-specific condition: [− 0.32, 0.11]). Next, to investigate how well-calibrated participants in each condition were, we calculated *c* values when confidence was categorized into three bins, *c* values range from 0 to 1 with lower scores meaning stronger calibration between confidence and accuracy. For participants in the eyewitness instructions condition (3 bins), *c* = 0.02, 95% CI [0.002, 0.05], for participants in the non-specific instructions condition (3 bins), *c* = 0.06, 95% CI [0.01, 0.09]. *ANDI* values were also calculated to investigate whether participants were able to discriminate between situations in which they were correct or incorrect through confidence. For participants in the eyewitness instructions condition (3 bins), *ANDI* = 0.002, 95% CI [− 0.007, 0.012], for participants in the non-specific instructions condition (3 bins), *ANDI* = 0.01, 95% CI [− 0.055, 0.053]. These low ANDI values indicate that confidence was unable to discriminate between correct and incorrect identifications.

#### Self-report analyses

Eight chi-square tests were conducted with a Bonferroni correction designating a per-test alpha level of 0.00625 using JASP (JASP Team, 2022). Descriptive statistics are reported in the Additional file 1. Participants in the eyewitness condition were more likely to endorse the choice that the man in the video stole the car (56.2%, *n* = 326) than those in the non-specific condition (35.8%, *n* = 204; *X*^*2*^(3) = 50.13, *p* < 0.001), though many in both groups (eyewitness: 37.6%, *n* = 218; non-specific: 56.5%, *n* = 218) endorsed a choice describing the video as a man leaving work in his own car. Participants in the eyewitness condition were more likely to claim they expected the video to contain a crime (63.6%, *n* = 369) than those in the non-specific condition (2.3%, *n* = 13; *X*^*2*^(1) = 486.89, *p* < 0.001). Participants in the eyewitness condition were also more likely to claim they expected to complete a lineup after the video (60.3%,* n* = 350) than those in the non-specific condition (4.2%, *n* = 24; *X*^*2*^(1) = 412.12, *p* < 0.001). Participants in the eyewitness condition more often claimed that they altered the way they watched the video (eyewitness: 55%, *n* = 319; non-specific: 29.5%, *n* = 168; *X*^*2*^(1) = 76.33, *p* < 0.001) and that they attempted to memorize the face of the criminal (eyewitness: 38.4%, *n* = 223; non-specific: 9.1%, *n* = 52; *X*^*2*^(1) = 135.53, *p* < 0.001). An overwhelming majority of participants accurately recognized their original instructions at the end of the procedure (95%, *n* = 1093). There were no significant differences between the two groups regarding when they claimed to memorize the face, *X*^*2*^(5) = 4.93, *p* = 0.424, whether they claimed to memorize specific details of the video other than the face, *X*^*2*^(1) = 1.93, *p* = 0.165, and their own rating of how well they paid attention to the video, *X*^*2*^(3) = 3.60, *p* = 0.309. A 2 × 2 ANOVA testing whether accuracy differed based on a participant’s response to “Did you expect to see a lineup?” produced no significant results, “Did you expect…” answer main effect *F*(1,1145) = 0.08, *p* = 0.780, condition main effect *F*(1,1145) = 1.25, *p* = 0.263, interaction term *F*(1,1145) = 0.18, *p* = 0.669, all effect sizes were 0.001 or smaller. A 2 × 2 ANOVA testing whether choosing rates differed based on a participant’s response to “Did you expect to see a lineup?” produced no significant results, “Did you expect…” answer main effect *F*(1,1145) = 0.83, *p* = 0.363, condition main effect *F*(1,1145) = 0.53, *p* = 0.468, interaction term *F*(1,1145) = 0.82, *p* = 0.365, all effect sizes smaller than 0.001.

## Discussion

We examined the impact of pre-event instructions on lineup identifications and confidence in those decisions. Participants were given either non-specific instructions (‘watch this video…’) or eyewitness instructions that revealed that a crime and lineup would occur. Partial and full ROC curves were generated to test the hypothesis that participants who received eyewitness instructions would show better discriminability on the lineup task than those who received non-specific instructions. We found that discriminability did not significantly differ by pre-event instruction condition, though participants who received eyewitness instructions were more likely to choose someone from the lineup than participants who received non-specific instructions. Confidence-accuracy analyses tested the hypothesis that participants who received eyewitness instructions would be more overconfident than those who received non-specific instructions. The confidence analyses also allowed us to test our competing predictions about whether eyewitness instructions would lead to better or worse calibration than non-specific instructions. However, we found that confidence-accuracy calibration did not significantly differ between pre-event instruction conditions.

Participants who read eyewitness pre-event instructions were more likely to report expecting to see a crime and lineup, actually having seen a crime, and altering the way they viewed the video than participants who read non-specific pre-event instructions. Yet, the pattern of results was not wholly clear: despite 95% of participants recognizing the instruction they read at the start of the study when shown a selection of options at the end of the study, only 60% of those in the eyewitness instruction condition reported expecting a lineup. It may be that while participants in eyewitness studies read instructions and are aware of them, they do not necessarily register their meaning. This may partially explain the lack of differences we found in the instruction conditions. Overall, we found little impact of pre-event instructions on people’s performance at identifying a culprit from a lineup. These findings have some positive implications for the literature on eyewitness identification. However, that instructions did not substantially change cognition in this study may display a need for future research on whether, when, and how people are able to adapt their conscious cognition to a novel task, even when given clear instructions.

### Instructions and discriminability

The hypothesis that participants who received eyewitness instructions would have higher discriminability than participants who received non-specific instructions was not supported. Thus, it seems that the effects of instructions on attention (Varakin & Hale, 2014), encoding strategy (Coin & Tiberghien, 1997; Craik & Tulving, 1975), or metacognition (Cox et al., 2021; Mazzoni & Nelson, 1995) seen in more basic work did not extend to this eyewitness identification paradigm. This finding is in line with those of Yarmey (2004), but contrasts findings of increased eyewitness identification accuracy when participants were warned of an upcoming crime or lineup (Cowan et al., 2014; Lindsay et al., 1998; Wulff & Hyman, 2022). The risk of Type II error in this experiment exists but is low, because this study was adequately statistically powered. A series of metacognitive explanations are perhaps more intriguing.

When provided with instructions or a strategy, people sometimes exhibit evidence of attempting to use the strategy without the expected concomitant increase in performance (see Bjorkland et al., 1997 for review). Our study design does not allow for us to test for utilization deficiencies, but the increase in choosing without an increase in discriminability that we observed is analogous to the decreased performance after training that researchers have observed in utilization deficiency studies (Bray et al., 1985; DeMarie-Dreblow & Miller, 1988). This utilization deficiency account suggests that our participants attempted to make use of the instructions to improve performance but that their efforts resulted in no changes in performance or even a decline in performance.

Related to the utilization deficiencies hypothesis, people’s meta-cognitive strategies for attention allocation and memorizing faces may be ineffective. It could be the case that participants’ individual differences in attention allocation are too strong for instructions to have an effect over and above their trait abilities (Draheim et al., 2022). People study specific features on faces when intentionally memorizing them, which does not align with the holistic manner in which faces are naturalistically encoded (Farah et al., 1998). If eyewitness instruction participants deployed a feature-based study strategy toward the culprit’s face, this could explain the lack of difference between conditions. Perhaps participants in the eyewitness instruction condition attempted to perform well but were limited in their ability to improve by their own metacognitive awareness and the difficulty of the task.

Finally, our study conditions provide another possible explanation for our findings. We used a short exposure duration in this study to create difficult witnessing conditions. However, it could be the case that the other conditions of the study and video were so simple that differences did not occur between the conditions. The simplicity of the event may determine whether attention instructions affect identification. As with many eyewitness identification studies, our video included only the culprit. Participants may easily focus on that single person. With more complex events, attention may become more important for focusing on individuals and for identification (Clifford & Hollin, 1981; Greene et al., 2017).

### Decision criterion

Participants in the eyewitness condition were more likely than participants in the non-specific condition to choose someone from the lineup, regardless of culprit presence though the overall effect was small. Perhaps participants in the eyewitness instruction condition believed their memory to be stronger or felt more pressure to make an identification compared to participants in the non-specific instruction condition and, instead of manifesting in increased discriminability, this manifested in increased choosing.

An alternative explanation is that participants in the eyewitness instruction condition believed themselves to have weaker memory traces than the participants in the non-specific condition. This may seem counterintuitive, as participants who received instructions should have had stronger memory traces and thereby stronger confidence in their memory than participants who did not. However, it is possible that participants who received instructions were either more aware of or surprised by their weak memory traces than participants who did not receive instructions. Research on autobiographical memory has found that participants typically subconsciously compensate for weaker memory traces in an incidental encoding condition by adopting a more liberal decision criterion (Popov & Dames, 2022). Similarly, Brewer et al. (2022) found that participants who infer that their memory trace is weak adopt a more lenient response criterion for identification decisions than participants who infer that their memory trace is strong. Future research is needed to understand how attempts to strengthen memory that do not manifest in stronger memory affect participants' inferences about their memory strength and criterion setting. The possibility of inclusion of an innocent bystander in a real-world lineup further complicates these matters (Wixted & Mickes, 2015; Wulff & Hyman, 2022).

### Confidence-accuracy calibration

We found no evidence for our hypothesis that eyewitness pre-event instructions may impact the calibration of confidence and accuracy as compared to non-specific pre-event instructions. Researchers have been attempting to decode the confidence-accuracy relationship for decades. Most recently, Wixed and Wells’s (2017) influential paper found that confidence and accuracy are well calibrated under what they called ‘pristine’ lineup procedure conditions. However, several studies have found that this relationship does not hold when encoding conditions are poor (e.g., Colloff et al., 2016; Giacona et al., 2021; Grabman et al., 2019; Lockamyeir et al., 2020; Seale-Carlisle et al., 2019; Semmler et al., 2018) though others show that it does (e.g., Semmler, et al., 2018). In the present study, participants were fairly well calibrated (analysis of *c*), but there were no significant differences between conditions (*OU* analyses), and confidence ratings did not discriminate between correct and incorrect responses well (ANDI). As can be seen from the two CAC graphs (of different bin sizes), most responses were made with medium confidence, and we see from the *ANDI* values that confidence discrimination was near or at floor levels. Furthermore, the bins only contained enough data for meaningful analyses after we dropped from 7 bins to 3, showing again the importance of having large datasets for these analyses to be useful. The need for such large samples emphasizes the applied question of the utility of any one witness’s confidence level (Sauer et al., 2019).

### Participants’ perceptions

We gathered a variety of self-report responses to measure how participants perceived the event and how the instructions impacted their expectations and behavior in the study. Of note, we were most interested in whether participants who received eyewitness instructions would report different attention or encoding strategies than participants who received non-specific instructions. The data showed that participants in the eyewitness condition were more likely than those in the non-specific instructions condition to endorse choices showing that they knew what to expect in the video and lineup and that they watched the video differently than they would have without the instructions by attempting to memorize the face of the criminal. Despite this, a substantial number of participants in the eyewitness instruction condition did not report doing anything differently than the non-specific instruction condition. The participants given eyewitness instructions did not claim to have memorized the face of the criminal earlier or to have paid more attention to the video than those given non-specific instructions. Most of these differences serve as evidence in favor of the functionality of our manipulation, but it is interesting to note that some people in the eyewitness condition reported that they did not expect a crime (36.4%, *n* = 211), did not expect a lineup (39.7%, *n* = 230), and did not see a crime (43.8%, *n* = 254) at the end of the study despite having transcribed instructions informing them of exactly that.^10^ That some participants reported they did not see a crime suggests they did not believe the event they witnessed was a criminal act. The current study used a video in which a man took a set of keys and started and drove off in a car, and it is reasonable for a viewer to interpret that they were his keys and his car (although the participants in the eyewitness instructions conditions were told otherwise). Even so, this leads us to recommend asking participants whether they believed the video they viewed was a crime, as that difference in perception could change how they think about the event as it transpires as well as their approach to any following memory tests. From Hyman and colleagues' (2021) work, we know that people do not always notice a crime occurring in their midst and that they sometimes confabulate details they did not witness.

### Recommendations for best practices in research

We have provided preliminary evidence that pre-event instructions may not affect lineup discrimination but that they may affect participants’ decision criterion, reported expectations, and efforts in an eyewitness paradigm. There are several reasons researchers may choose to be cautious about using revealing instructions despite the null effect we found of instructions on discriminability. First, this research should serve as a starting point (alongside Wulff & Hyman, 2022) for understanding the impact of pre-event instructions on eyewitness memory. Further research is needed before strong conclusions can be drawn about the impact of pre-event instructions. It remains possible that our participants did not fully appreciate the implication of the instruction they received. A critical question is whether this is representative of people or due to the unique combination of viewing conditions in our study. Second, revealing instructions are not representative of most real world eyewitness conditions. As eyewitness research aims to generalize to real world circumstances, it is important that research be realistic.

Our systematic review found that researchers rarely report pre-event instructions. Reporting enough methodological detail so that an independent researcher could replicate a study is crucial to advancing scientific progress. We therefore recommend that pre-event instructions—and the wording used on participant recruitment and information materials—should be reported clearly, fully, and transparently in published research. Importantly, had instructional details been reported in the literature, a meta-analysis could have been conducted to determine the effect of instructions on eyewitness identification and confidence.

### Future directions

We do not yet know whether instructions have an impact under different witnessing conditions or whether instructions may interact with other variables. A particularly important potential covariate is scene complexity. We found limited effects of instructions in the current study when scene complexity was low. Instructions may have a larger impact when witnessing conditions are more complex because participants must make decisions about where to orient their attention. Murphy, Greene, and colleagues have found that eyewitnesses under high perceptual load remember less, are less likely to identify a person in the periphery of an event, and are more likely to experience change blindness for a person than eyewitnesses under low perceptual load (Murphy & Greene, 2016; Murphy & Murphy, 2018).

From Wulff and Hyman’s (2022) results and those of the current study, a pattern is emerging in which we see that witnesses' metacognition may be so poor that a non-trivial number of people do not know how to control their encoding processes even after they are told that a crime is coming and they will later see a lineup. These participants are nonetheless willing to offer decisions on lineups and are sometimes quite confident. Developing a clearer understanding of these witnesses, and their behavior under realistic witnessing conditions, may be our most important future research.

## Conclusions

We hypothesized that revealing pre-event instructions would lead to higher discriminability compared to non-revealing instructions and that instructions would impact confidence-accuracy calibration. We found no support for our hypotheses, but instructions had a small effect on choosing and participants’ reported strategy toward engaging with the study. We call on researchers to think carefully about all of their methodological decisions and to enhance their methodological reporting and transparency.

## Supplementary Information

**Additional file 1:** Descriptive tables for additional self-report measures, Traditional CAC plots.

## Data Availability

Data and materials are available on the Open Science Framework (osf.io/zb85d).

## Footnotes

1 Note that we wrote “or” rather than “and” as it is the more inclusive term in searching algorithms, and we wished to capture all instances of studies that used the term lineup, the term eyewitness identification, or both.

2 Some other discoveries were interesting but are outside the purpose of this manuscript. Almost every crime in every stimulus video involves a theft of some kind. Most papers report the length of their video or the length of the exposure to the culprit, but not always both. None of the papers provide direct access to their video(s).

3 Note that these values already account for CA and CP lineup groups, as both are required to create ROC curves.

4 We thank Melissa Colloff and John Wixted for sharing their sample size calculation tool.

5 The Stage 1 Report describes a plan for a scale from 50 to 100%, but a programming error left the experiment’s confidence scale at the 1–7 scale used for a previous study. This scale difference does not fundamentally change any conclusions or analyses.

6 The Stage 1 report described an identity question “How old are you?” and ‘bot’ questions (e.g. Please select ‘strongly agree’ from the choices below.) which were respectively altered and removed to be more interesting to participants.

7 Some participants violated more than one exclusion criteria, thus the total of these *N*’s is higher than the number of people excluded.

8 Widening bin size to move from 7 bins to 3 was planned in our Stage 1 Report, though we changed from a scale of 50–100 to a scale of 1–7 due to a programming error that did not update the scale from a previous study.

9 Boekaerts and Rozendaal’s paper does an excellent job of explaining each of these statistics upon which we could not possibly improve, thus we refer interested readers to their paper.

10 There is, of course, the possibility that some participants disbelieved the instructions from the beginning, but some pilot data that showed 80 of 100 participants believed that the video would show a crime before it began, so this possibility only partially explains the percentages cited above. These responses were to “Before the video started, did you believe that the video would feature a crime? Note that we aren't asking whether you think the crime was real, just whether you believed that a crime would be shown,” meaning that participants’ impressions may have changed during the video itself. This change would not explain the differences in choosing, however, as we would assume that a participant who felt doubtful about the criminality of the activities in the video would become less likely to choose, not more.
