# Correction: The effect of pre-event instructions on eyewitness identification

**DOI:** 10.1186/s41235-023-00489-8

**Published:** 2023-05-29

**Authors:** Mario J. Baldassari, Kara N. Moore, Ira E. Hyman, Lorraine Hope, Eric Y. Mah, D. Stephen Lindsay, Jamal Mansour, Renan Saraiva, Ruth Horry, Hannah Rath, Lauren Kelly, Rosie Jones, Shannan Vale, Bethany Lawson, Josh Pedretti, Tomás A. Palma, Francisco Cruz, Joana Quarenta, Ine Van der Cruyssen, Mila Mileva, Jessica Allen, Brittany Jeye, Sera Wiechert

**Affiliations:** 1 University of Saint Francis, 2701 Spring Street, Fort Wayne, IN 46808 USA; 2 Oklahoma State University, 116 Psychology Building, Stillwater, OK 74074 USA; 3 Western Washington University, 516 High Street, Bellingham, WA 98225 USA; 4 University of Portsmouth, King Henry I Street, PortsmouthHampshire, PO1 2DY UK; 5 University of Victoria, STN CSC, PO Box 1700, Victoria, BC V8W 2Y2 Canada; 6 University of Lethbridge, 4401 University Drive, Lethbridge, AB T1K 3M4 Canada; 7 Swansea University, Singleton Park, Sketty, Swansea, SA2 8PP UK; 8 CICPSI, Faculdade de Psicologia, Universidade de Lisboa, Cidade Universitária, Alameda da Universidade, 1649-004 Lisbon, Portugal; 9 University of Amsterdam, 1012 WX Amsterdam, Netherlands; 10 University of Plymouth, Drake Circus, Plymouth, PL4 8AA UK; 11 Worcester State University, 486 Chandler St, Worcester, MA 01602 USA

**Correction: Cognitive Research: Principles and Implications (2023) 8:16** **https://doi.org/10.1186/s41235-023-00471-4**

The original article [1] contained an obsolete email address for the Corresponding Author, Mario J. Baldassari, as well as a minor typo in Sera Wiechert’s name. Both have since been corrected.

