# Supplementary material for: The effect of pre-event instructions on eyewitness identification
Source: Cogn Res Princ Implic. 2023 Feb 28;8:16. doi: 10.1186/s41235-023-00471-4 (PMC9975131; doi:10.1186/s41235-023-00471-4)

Descriptive tables for self-report measures

| **Condition** | | **Video Content** | | **Frequency** | | **Percent** |  | |
| --- | --- | --- | --- | --- | --- | --- | --- | --- |
| Eyewitness |  | Don't know |  | 35 |  | 6.03 |  |  |
|  |  | Guy argued with a friend |  | 1 |  | 0.17 |  |  |
|  |  | Guy grabbed his keys and went to leave work |  | 218 |  | 37.59 |  |  |
|  |  | Guy grabbed keys off a desk and stole the car they were for |  | 326 |  | 56.21 |  |  |
|  |  | Total |  | 580 |  | 100 |  |  |
| Non-specific |  | Don't know |  | 36 |  | 6.33 |  |  |
|  |  | Guy argued with a friend |  | 2 |  | 0.351 |  |  |
|  |  | Guy grabbed his keys and went to leave work |  | 327 |  | 57.47 |  |  |
|  |  | Guy grabbed keys off a desk and stole the car they were for |  | 204 |  | 35.85 |  |  |
|  |  | Total |  | 569 |  | 100 |  |  |

| **Condition** | | **Which Instruction?** | | **Frequency** | | **Percent** |  | |
| --- | --- | --- | --- | --- | --- | --- | --- | --- |
| Eyewitness |  | Don’t know |  | 2 |  | 0.34 |  |  |
|  |  | Watch this video of a crime. You will be asked if you can identify the criminal from a lineup later. |  | 540 |  | 93.10 |  |  |
|  |  | Watch this video. |  | 38 |  | 6.55 |  |  |
|  |  | Total |  | 580 |  | 100 |  |  |
| Non-specific |  | Don’t Know |  | 5 |  | 0.88 |  |  |
|  |  | Watch this video of a crime. You will be asked if you can identify the criminal from a lineup later. |  | 11 |  | 1.93 |  |  |
|  |  | Watch this video. |  | 553 |  | 97.19 |  |  |
|  |  | Total |  | 569 |  | 100 |  |  |

| **Condition** | | **Did you expect a crime?** | | **Frequency** | | **Percent** | |  |
| --- | --- | --- | --- | --- | --- | --- | --- | --- |
| Eyewitness |  | No |  | 211 |  | 36.38 |  |  |
|  |  | Yes |  | 369 |  | 63.62 |  |  |
|  |  | Total |  | 580 |  | 100 |  |  |
| Non-specific |  | No |  | 556 |  | 97.72 |  |  |
|  |  | Yes |  | 13 |  | 2.28 |  |  |
|  |  | Missing |  | 0 |  | 0.00 |  |  |
|  |  | Total |  | 569 |  | 100 |  |  |

| **Condition** | | **Did you expect a lineup?** | | **Frequency** | | **Percent** | |  |
| --- | --- | --- | --- | --- | --- | --- | --- | --- |
| Eyewitness |  | No |  | 230 |  | 39.66 |  |  |
|  |  | Yes |  | 350 |  | 60.34 |  |  |
|  |  | Total |  | 580 |  | 100 |  |  |
| Non-specific |  | No |  | 545 |  | 95.78 |  |  |
|  |  | Yes |  | 24 |  | 4.22 |  |  |
|  |  | Total |  | 569 |  | 100 |  |  |

| **Condition** | | **Did you alter your attention?** | | **Frequency** | | **Percent** | |  |
| --- | --- | --- | --- | --- | --- | --- | --- | --- |
| Eyewitness |  | No |  | 261 |  | 45.00 |  |  |
|  |  | Yes |  | 319 |  | 55.00 |  |  |
|  |  | Total |  | 580 |  | 100 |  |  |
| Non-specific |  | No |  | 401 |  | 70.48 |  |  |
|  |  | Yes |  | 168 |  | 29.52 |  |  |
|  |  | Total |  | 569 |  | 100 |  |  |

| **Condition** | | **Did you try to memorize the face?** | | **Frequency** | | **Percent** | |  |
| --- | --- | --- | --- | --- | --- | --- | --- | --- |
| Eyewitness |  | No |  | 357 |  | 61.55 |  |  |
|  |  | Yes |  | 223 |  | 38.45 |  |  |
|  |  | Total |  | 580 |  | 100 |  |  |
| Non-specific |  | No |  | 517 |  | 90.86 |  |  |
|  |  | Yes |  | 52 |  | 9.14 |  |  |
|  |  | Total |  | 569 |  | 100 |  |  |

| **Condition** | | **When did you decide to memorize the face?** | | **Frequency** | | **Percent** | |  |
| --- | --- | --- | --- | --- | --- | --- | --- | --- |
| Eyewitness |  | As soon as he rounds the corner |  | 28 |  | 4.83 |  |  |
|  |  | Before the video began |  | 93 |  | 16.03 |  |  |
|  |  | In the final moments, when he starts the car |  | 61 |  | 10.52 |  |  |
|  |  | Other |  | 8 |  | 1.38 |  |  |
|  |  | When he leaves the office |  | 13 |  | 2.24 |  |  |
|  |  | When he steals the keys |  | 20 |  | 3.45 |  |  |
|  |  | Missing |  | 357 |  | 61.55 |  |  |
|  |  | Total |  | 580 |  | 100 |  |  |
| Non-specific |  | As soon as he rounds the corner |  | 8 |  | 1.41 |  |  |
|  |  | Before the video began |  | 14 |  | 2.46 |  |  |
|  |  | In the final moments, when he starts the car |  | 17 |  | 2.99 |  |  |
|  |  | Other |  | 4 |  | 0.70 |  |  |
|  |  | When he leaves the office |  | 4 |  | 0.70 |  |  |
|  |  | When he steals the keys |  | 5 |  | 0.88 |  |  |
|  |  | Missing |  | 517 |  | 90.86 |  |  |
|  |  | Total |  | 569 |  | 100 |  |  |

| **Condition** | | **Did you try to memorize other details?** | | **Frequency** | | **Percent** | |  |
| --- | --- | --- | --- | --- | --- | --- | --- | --- |
| Eyewitness |  | No |  | 198 |  | 34.138 |  |  |
|  |  | Yes |  | 381 |  | 65.690 |  |  |
|  |  | Missing |  | 1 |  | 0.172 |  |  |
|  |  | Total |  | 580 |  | 100.000 |  |  |
| Non-specific |  | No |  | 217 |  | 38.137 |  |  |
|  |  | Yes |  | 352 |  | 61.863 |  |  |
|  |  | Missing |  | 0 |  | 0.000 |  |  |
|  |  | Total |  | 569 |  | 100.000 |  |  |

| **Condition** | **How much attention did you pay to the video?** | | | | | | | **Frequency** | | | | | **Percent** |
| --- | --- | --- | --- | --- | --- | --- | --- | --- | --- | --- | --- | --- | --- |
| Eyewitness | A lot |  |  |  |  |  |  | 140 |  |  |  |  | 24.138 |
|  | Moderate |  |  |  |  |  |  | 309 |  |  |  |  | 53.276 |
|  | Somewhat |  |  |  |  |  |  | 125 |  |  |  |  | 21.552 |
|  | Not at all |  |  |  |  |  |  | 6 |  |  |  |  | 1.034 |
|  | Total |  |  |  |  |  |  | 580 |  |  |  |  | 100.000 |
| Non-specific | A lot |  |  |  |  |  |  | 112 |  |  |  |  | 19.684 |
|  | Moderate |  |  |  |  |  |  | 314 |  |  |  |  | 55.185 |
|  | Not at all |  |  |  |  |  |  | 6 |  |  |  |  | 1.054 |
|  | Somewhat |  |  |  |  |  |  | 137 |  |  |  |  | 24.077 |
|  | Total |  |  |  |  |  |  | 569 |  |  |  |  | 100.000 |

Traditional CAC plots

Figure 1: 7-bin Traditional CAC


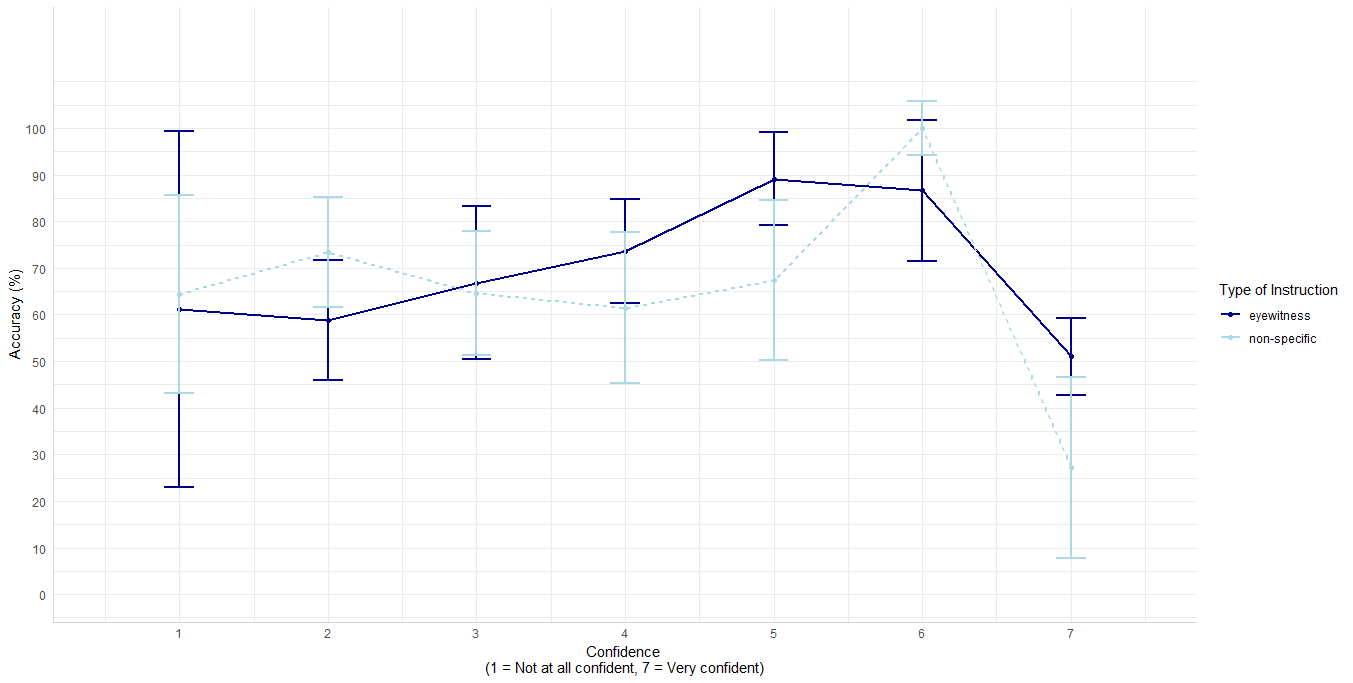


Figure 2: 3-bin Traditional CAC


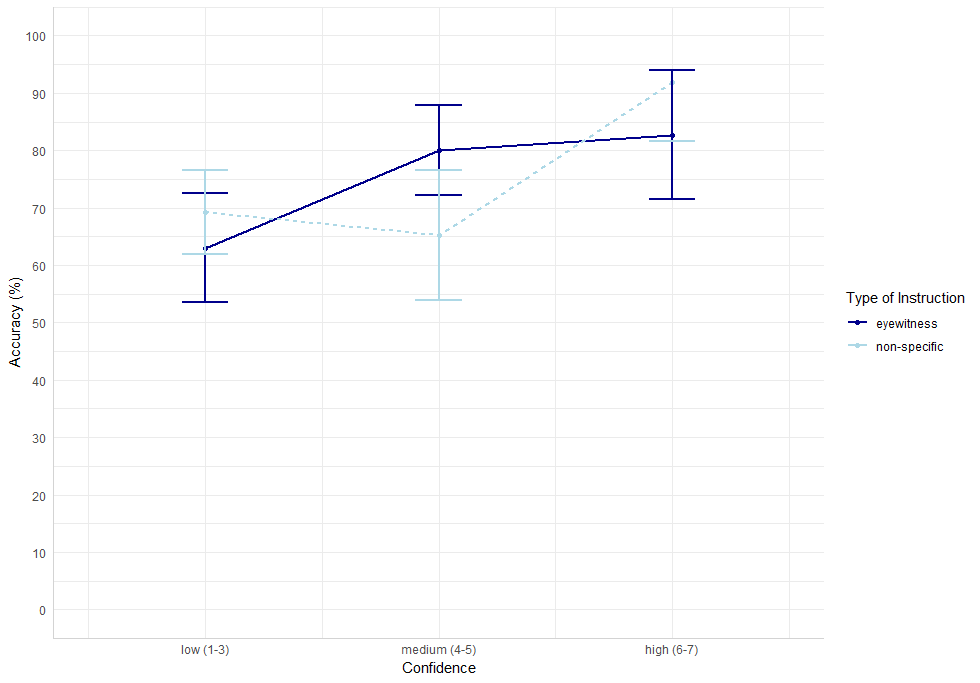

Supplement: Supplementary file 1 — Additional file 1: Descriptive tables for additional self-report measures, Traditional CAC plots. [file 41235_2023_471_MOESM1_ESM.docx]
